# Effects of extreme rainfall events are independent of plant species richness in an experimental grassland community

**DOI:** 10.1007/s00442-019-04476-z

**Published:** 2019-08-10

**Authors:** Francisco M. Padilla, Liesje Mommer, Hannie de Caluwe, Annemiek E. Smit-Tiekstra, Eric J. W. Visser, Hans de Kroon

**Affiliations:** 1 Department of Experimental Plant Ecology, Institute for Water and Wetland Research, Radboud University Nijmegen, PO Box 9010, 6500 GL Nijmegen, The Netherlands; 2 Department of Agronomy, University of Almería, La Cañada, 04120 Almería, Spain; 3 Plant Ecology and Nature Conservation Group, Wageningen University, P.O. box 47, 6700 AA Wageningen, The Netherlands

**Keywords:** Climate change, Drought, Biodiversity, Overyielding, Stability, Resistance, Roots

## Abstract

Global climate models predict more frequent periods of drought stress alternated by heavier, but fewer rainfall events in the future. Biodiversity studies have shown that such changed drought stress may be mitigated by plant species richness. Here, we investigate if grassland communities, differing in species richness, respond differently to climatic extremes within the growing season. In a 3-year outdoor mesocosm experiment, four grassland species in both monoculture and mixture were subjected to a rainfall distribution regime with two levels: periods of severe drought in the summer intermitted by extreme rainfall events versus regular rainfall over time. Both treatments received the same amount of water over the season. Extreme rainfall combined with drought periods resulted in a 15% decrease in aboveground biomass in the second and third year, compared to the regular rainfall regime. Root biomass was also reduced in the extreme rainfall treatment, particularly in the top soil layer (− 40%). All species developed higher water use efficiencies (less negative leaf δ^13^C) in extreme rainfall than in regular rainfall. These responses to the rainfall/drought treatment were independent of species richness, although the mixtures were on an average more productive in terms of biomass than the monocultures. Our experimental results suggest that mixtures are similarly able to buffer these within-season rainfall extremes than monocultures, which contrasts with findings in the studies on natural droughts. Our work demonstrates the importance of investigating the interactions between rainfall distribution and drought periods for understanding effects of climate change on plant community performance.

**Electronic supplementary material:**

The online version of this article (10.1007/s00442-019-04476-z) contains supplementary material, which is available to authorized users.

## Introduction

There has been growing attention in the scientific literature to increasing frequency of extreme climatic events (Easterling et al. 2000; IPCC 2014; Mann et al. 2017). Most global climate models forecast changes in precipitation in the future (IPCC 2014; Trenberth 2011), which involves changes in both the frequency and magnitude of the events within the growing season (Frei et al. 1998; IPCC 2014; Min et al. 2011). More intense rain events, fewer rain days, and longer droughts are forecasted in high latitude areas of Europe (Beniston et al. 2007; Kovats et al. 2014). It is, therefore, likely that plants will experience more frequent periods of drought stress alternated by extreme rainfall events in the future (Knapp et al. 2008; Weltzin et al. 2003).

How grasslands will respond to such extreme combinations of intense rainfall and drought within the growing season is poorly understood. Previous research suggests that the combination of intense rainfall and drought periods within the growing season can lead to reduced aboveground biomass (Gibson-Forty et al. 2016; Heisler-White et al. 2009; Knapp et al. 2002), at least in mesic grasslands (Knapp et al. 2008). However, in semi-arid grasslands, Heisler-White et al. (2009) found that aboveground biomass increased, rather than decreased. From these studies it is clear that effects of climate change cannot simply be studied by comparing the effects of different amounts of rainfall on plant growth. Variation in both the intensity of precipitation events and the period of time between those events (i.e., the drought severity) is equally important (Craine et al. 2012). Also in pot experiments where the total amount of water was left unchanged, root growth was found to be very sensitive to the frequency of water supply (Maestre and Reynolds 2007; Padilla et al. 2013) with less roots produced under less frequent watering (Gibson-Forty et al. 2016). The roots likely play a crucial role (Fort et al. 2017; Hoekstra et al. 2015), as they are responsible for the uptake of water from extreme watering events, as well as from deeper soil layers in intermittent periods of drought.

Considerable research effort has been devoted to the question how plant biodiversity mediates plant community responses to extreme rainfall (e.g., Isbell et al. 2015; Vogel et al. 2012; Wright et al. 2015). Biodiversity has positive effects on biomass production (referred to as overyielding, i.e., higher biomass of species mixtures than the average biomass of monocultures (Cardinale et al. (2011); Marquard et al. (2009); Mommer et al. (2010)). Evidence is accumulating that biodiversity also increases the buffering capacity of ecosystems to climatic extremes [i.e., maintain biomass production under drought, e.g., Isbell et al. (2015), Tilman and Downing (1994)]. Most of these studies consider natural variation in precipitation over the years, rather than variation of precipitation within the season. For example, in a meta-analysis on grassland biodiversity experiments, ranging from 1 to 60 species, Isbell et al. (2015) showed that communities with higher species richness are more resistant to drought than low diverse communities, as biomass decline in extreme years was less in communities with high compared to low species richness. It has been suggested that these biodiversity effects can be explained by the so called ‘insurance hypothesis’(Yachi and Loreau 1999). Communities that are more diverse are more likely to buffer climatic fluctuations because species may compensate for each other as they differ in their responses to climatic events. By chance, species-rich communities may contain more species that maintain functioning under extreme conditions. Specifically, diverse plant communities are more likely to contain plant species with higher drought tolerance than low diverse communities. Drought tolerance can be reflected by the ability to survive under long periods of drought, but also by the ability to root deeper, as this leads to being able to profit from extreme water events that saturate the water profile (De Boeck et al. 2006; Guderle et al. 2018; Heisler-White et al. 2008; Hoekstra et al. 2015). Indeed, there is evidence for lower water-use efficiency in more diverse communities compared to monocultures (Guderle et al. 2018; Van Peer et al. 2004) because plants in these communities had access to more water and, thus, increased stomatal conductance. Following the insurance hypothesis, such diverse communities should be better able to buffer precipitation extremes within the season than monocultures.

Here, we designed an experiment in which total amount of rainfall was similar over the growing season, but the intensity of rainfall events varied together with the length of the intermittent periods without rain (i.e., drought). In particular, we compared an extreme watering treatment throughout the season, where spring and summer periods of at least 4 weeks of drought were alternated with heavy rainfall events, to a regular rainfall treatment where plants were watered every other day. We investigated experimentally how plant communities responded to within-season rainfall extremes in terms of biomass production and root allocation patterns, and whether there were differences in response between a four-species mixture and the respective monocultures. Although conducted over a relatively narrow diversity gradient (1–4 species only), our study may provide insights into the consequences of climate change on grasslands in general, as previous studies showed that responses of plants over a biodiversity gradient of one to four species were indicative of biodiversity effects in general (e.g., Isbell et al. 2015; Marquard et al. 2009). The experimental treatments were imposed over three successive growing seasons in mesocosms under outdoor conditions.

The responses of four species in monoculture, two more shallow-rooted grasses, and two deeper-rooting forbs were compared with the four-species mixture. To test whether species mixtures were more resistant to within-season water fluctuations than monocultures, and to obtain a better understanding of the belowground mechanisms, we (1) quantified aboveground and belowground biomass of monocultures and the mixture over the 3 years of study. At the same time, we (2) tested whether the root distribution shifted to deeper layers under the extreme rainfall, and whether this shift was different in the mixture than in monocultures. Then we (3) quantified differences in water-use efficiency (WUE; as leaf δ^13^C) between the species in monocultures and the mixture, to examine whether differences in drought tolerance contributed to resistance to the rainfall extreme.

## Materials and methods

### Species and experimental design

Two C3-grass species, *Anthoxanthum odoratum* L. and *Festuca rubra* L., and two dicots, *Centaurea jacea* L. and *Plantago lanceolata* L., were used in an experiment conducted from 2010 to 2013 in the Phytotron of the Radboud University (Nijmegen, The Netherlands). These species have been used regularly in previous biodiversity studies (Cong et al. 2014; Mommer et al. 2010, 2018), with the two grasses as the shallow and the two forbs as the deeper rooting species in these communities. The species are common hay meadow species in mid-western Europe. In the Netherlands, the four species often co-occur in mesic meadows, occurring in a temperate climate (yearly precipitation approximately 800 mm, mean day temperature 14 °C). The Nijmegen Phytotron is an outdoor mesocosm facility (35 × 12 m; 32 containers each holding three mesocosm units) that consists of a 4-m tall tunnel frame with a transparent polyethylene cover that acts as a rain shelter and open at each longitudinal side except for an insect screening mesh. Daily air temperature and relative air humidity were, on an average, 0.4 °C and 2.2% higher, respectively, inside the phytotron compared to outside (June 2008 to August 2009 period), regardless of the season.

The research was conducted in 48 separate mesocosm units of 50(*w*) × 50(*l*) × 70(*h*) cm each, grouped by 3 in 16 polyethylene containers. The containers were insulated with thick polyisocyanurate insulation boards to prevent heating by irradiation and were placed on a concrete bench constructed below soil surface, so that the top of the containers was at the level of the surrounding soil outside the phytotron. Each unit had a separate drainage outlet at the bottom.

Each unit had a 5-cm layer of coarse gravel at the bottom and was filled with 60 cm of a mix of nutrient-rich and nutrient-poor sand (1:3 volume, respectively). The loamy-sandy soil (88.3 ± 1.4% sand, 4.0 ± 0.5% silt; bulk density 1.47 ± 0.01 kg L^−1^) contained 10 ± 0.2 g kg^−1^ organic matter (OM), 2.1 ± 0.05 g kg^−1^ total nitrogen (N), 37 ± 3 mg kg^−1^ nitrate (NO_3_^−^), 0.7 ± 0.04 mg kg^−1^ ammonium (NH_4_^+^), and 57.4 ± 1 mg kg^−1^ phosphate (PO_4_^3−^). Approximate values for soil saturation (42.1 ± 0.4%, volumetric), field capacity (9.4 ± 1.0%), and permanent wilting point (4.9 ± 0.8%) were estimated from soil texture and organic matter content, using the Soil Water Characteristics program (Saxton and Rawls 2006). The top 5 cm of the unit was left unfilled to prevent water spilling after large irrigation events.

Either monocultures of each of the four species or mixtures of the four species (1:1:1:1 proportion) were established in each unit in September 2010 in a substitutive design. Thirty-six seedlings (6 × 6) were raised from local seeds collected from the forelands of the river Rhine near Nijmegen (The Netherlands), and then transplanted in each unit, resulting in a plant density of 144 m^−2^. The inner area of 4 x 4 plants (32 x 32 cm) was used for further measurements to avoid edge effects. Interplant distant of inner plants was 8 cm. All plots were carefully weeded if necessary.

The experiment consisted of four blocks, each with one replicate of the monocultures and two of the mixtures, and one replicate of each of the two rainfall treatments.

Rainfall treatments were based on precipitation records of the nearest meteorological station (Supplementary Material) and on climate change scenarios for mid-western Europe of increased extreme rain events interspersed with longer dry periods (IPCC 2014; Kovats et al. 2014; van der Hurk et al. 2006). Following long-term precipitation averages in this part of the Netherlands (see Supplementary Material), the regular rainfall treatment consisted of one watering event appr. every other day of 4.8 mm (Table 1; for the last 2 years of the experiment). In the extreme rainfall treatment, drought periods of an average of 30 days were imposed, similar in length to the dry periods in the experiment of De Boeck et al. (2011), based on climate change predictions for mid-western Europe (IPCC 2014; Kovats et al. 2014; van der Hurk et al. 2006). Watering every 30 days in the extreme treatment totalled the same amount of water provided in the regular rainfall treatment over that period (on average 69 mm for the last 2 years of the experiment; Table 1). Rainfall events of this extent per 1 or 2 days are getting increasingly common in Western Europe and other parts of the world. The extreme watering was split over three consecutive days to prevent water spilling. Rainfall treatments were applied only in the growing season (Mar–Apr to Sep), consistent with forecasted rainfall scenarios, with a total of six dry spells in 2011 and 2012, and five in 2013. In autumn and winter, the same watering was applied to both treatments once or twice a week. Rainfall in the first year deviated from the remaining years because due to transpiration by the high aboveground biomass, soils quickly dried out to wilting point values. Water supply in the first year was, thus, increased to nearly 1.5 times higher than in the second and third years, similarly in the regular and extreme watering treatments.

**Table 1:** Average air temperatures (24 h minimum, mean, and maximum), spring and summer rainfall, and reference evapotranspiration (ETo) at the nearest meteorological station (Volkel, 23 km from the Nijmegen Phytotron, 51°39′N, 05°42′W, 20 m above sea level, KNMI)

Year	Temperature (°C)^a^			Rainfall (mm)^a^	ET_0_ (mm)^a^	Period of dry spells and extreme rainfall	Rainfall treatment	Number of dry spells and duration	Rainfall (mm)	Average rain event (mm)	Number of rain events	Drainage (mm)^b^
	Min	Mean	Max									
2011	9.7	15.4	21.0	393.0	485.9	13 Apr–5 Oct	Regular	–	544.9	6.2	88	86.5 ± 3.1
							Extreme	6 (28 days)	544.9	90.8	6	69.1 ± 2.5
2012	9.3	14.6	19.8	484.1	478.1	14 Mar–3 Oct	Regular	–	448.9	4.8	93	78.7 ± 6.5
							Extreme	6 (33 days)	448.9	74.8	6	114.1 ± 4.4
2013	8.8	14.8	20.2	318.0	480.7	8 Apr–21 Sep	Regular	–	320.5	4.7	68	49.0 ± 4.4
							Extreme	5 (32 days)	320.5	64.1	5	67.2 ± 2.6

Characteristics of the two rainfall treatments as the number of dry spells and their average duration, the total amount of rainfall over the period of treatment in each of the years, the amount of rainfall per rain event, the number of rain events and the drainage. All rain and drainage amounts are given as mm (L m^−2^). In the extreme rainfall, each rain event consisted of watering during three consecutive days

^a^Period April to September in Volkel

^b^Values are averages of the five plant communities (four monocultures and one mixture)

Watering was provided early in the morning and was programmed with an automatic irrigation system (PRIVA, de Lier, The Netherlands). Drippers were evenly distributed in each mesocosm and stakes were inserted in the top soil of each unit. In winter, the irrigation system was removed for frost protection and the containers were watered manually with a garden sprayer. Rainwater collected in a near vessel was used for irrigation from April 2013 onwards; tap water of the Nijmegen municipality was used in the first 2 years of the experiment. During the experimental treatments, drainage from each experimental unit was collected every 2 days, to estimate how much water was lost to the deep subsoil.

### Soil moisture measurements

Soil moisture was continuously monitored during the experiment in one of the units of each plant species composition x rainfall treatment in monocultures, and in two of the units in mixtures (the average of the two being used throughout). At soil filling of the experimental units, soil moisture sensors (S1—soil probe, Applied Environmental Science Ltd, Green End, Cambridge, UK) were placed at two depths, vertically inserted in the 0–6 cm top soil layer, and placed horizontally at 35 cm depth in the middle layer. Readings were averaged daily in an automatic system (PRIVA).

Individual soil moisture measurements were made with a portable sensor (ThetaProbe ML2x, Delta-T Devices Ltd., Cambridge, UK) in all experimental units (*n* = 4 in monocultures, *n* = 8 in mixtures, for each plant species composition × rainfall treatment combination) on different dates (*n* = 15–23) during the growing season of each year, before and after watering events. These measurements allowed characterization of the effect of plant species composition and rainfall treatment on soil moisture with appropriate replication. Measurements were made in the 0–6 cm top layer, where most of the root mass of these plant communities concentrates (Mommer et al. 2010).

### Aboveground and belowground harvests

In early September of each of the 3 years, aboveground biomass was harvested in an inner 32 × 32 cm area by clipping shoots 1–2 cm above the soil surface, a common practice for hay meadows where these species occur (Mommer et al. 2010). In the mixture, plant material was sorted by species.

Belowground biomass was determined after shoot harvest by taking four replicated soil cores (20 mm diameter) in each unit, in the inner area, down to four soil layers (0–10, 10–20, 20–40, and 40–60 cm depth increments). The four cores were bulked per unit. In the mixture, the sampling point was between the positions where plants of the four species were planted. The holes were filled with substrate after coring. Different positions were sampled each year. Roots per soil layer were collected after carefully rinsing with tap water.

Shoot and root dry weights were determined after drying samples at 70 °C until constant weight. Total aboveground and belowground biomass was expressed as total dry weight relative to sampled area (g m^−2^). Root mass density per soil layer was calculated as dry weight relative to sampled soil volume (g dm^−3^).

### Leaf δ^13^C analysis

At the end of each growing season, well-lit healthy leaves from several plants located in the inner area of each unit were collected, bulked per unit in paper bags and immediately ovendried at 70 °C until constant weight for determination of δ^13^C. In the mixture, leaf samples were separated by species. Dry samples were ground to a fine powder, weighed, and tinned for isotopic analysis in a mass spectrometer (Finnigan DeltaPlus IRMS, Carlo-Erba, Thermo Fisher Scientific, Waltham, MA, USA) at the General Instrumentation department of the Faculty of Science of Radboud University (Nijmegen, The Netherlands). The isotopic abundance was expressed in delta notation (*δ*) in parts per thousand (‰) as *δ* = (*R*_sample_/*R*_standard_−1) × 1000, where *R*_sample_ and *R*_standard_ are the molar ratios of heavy to light isotope of the sample and the international standard, respectively.

Leaf δ^13^C provides a measure of water-use efficiency (WUE, i.e., carbon gain per water lost), with less negative δ^13^C values indicating higher WUE, that is, higher carbon production per unit of water loss (Dawson et al. 2002; Lambers et al. 2008).

### Data analysis

Data from portable soil moisture measurements were evaluated by repeated-measures analysis of variance (RM-ANOVA), using time (i.e., date of soil moisture measurements) as a within-subjects factor and block, plant community (four monocultures and one mixture) and rainfall treatment (regular and extreme rainfall) as between-subjects factors. Differences in drainage, aboveground, and belowground biomass and root mass density were analyzed with factorial ANOVA, with block, plant community, and rainfall treatment as factors. For root mass density, the soil layer was included as a factor. Finally, biomass and δ^13^C in the four-species mixtures were separately tested against the average of the monocultures (factor ‘diversity’), as in tests of overyielding (Hendriks et al. 2013; Mommer et al. 2010).

In the replacement design in the mixture, where plant density was equal to monocultures and each species accounted for ¼ of plant density in monocultures, aboveground and belowground overyielding was tested (i.e., if the observed values of aboveground and belowground biomass in the mixture exceeded those expected from the average of the respective monocultures). Factorial ANOVA was performed with block, mixture, and rainfall treatment as factors. Mixture consisted of observed mixture values versus expected mixture values, with expected mixture values calculated from monocultures as a sum of ¼ of each of the four monocultures. Comparing the observed mixture values to expected mixture values calculated from the average of the respective monocultures is a common procedure in biodiversity research (Hector et al. 2002; Loreau and Hector 2001; Roscher et al. 2005). The factor rainfall consisted of regular and extreme rainfall, respectively.

All statistical analyses were conducted for each year independently. When main effects or any interactions were statistically significant at *p* < 0.05, LSD post hoc tests were used for pair-wise comparisons. Data were transformed to meet ANOVA assumptions when necessary. To meet the Mauchly’s sphericity assumption of RM-ANOVA, Huynh-Feldt adjusted degrees of freedom was used for within-subject effects when epsilon was > 0.75; the Greenhouse–Geisser adjusted degrees of freedom was used when epsilon was < 0.75 (Girden 1992). As the block effect was not significant in the analysis, data were re-analyzed omitting this factor. Data are given as mean ± standard error throughout. All analyses were run with IBM SPSS Statistics v.22.0 (International Business Machines Corp., Armonk, NY, USA).

## Results

### Soil moisture and water balance

**Fig. 1:**
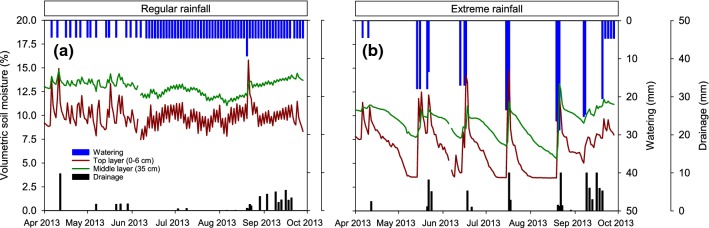
Soil moisture dynamics in the growing season of 2013 in the top (0–6 cm depth) and middle (35 cm depth) soil layers, and watering and drainage, in the regular (**a**) and extreme (**b**) rainfall treatments. Soil moisture values are daily averages of one replicate of each of the four monocultures and two replicates for the mixture. Soil moisture dynamics for years 2011 and 2012 are given in Figure S1. Saturation, field capacity, and wilting point of the soil are 42.1%, 9.4%, and 4.9%, respectively

The two rainfall regimes resulted in highly contrasting soil moisture dynamics in the top and middle soil layers over the growing season (Fig. 1; Figure S1). Under regular rainfall, soil moisture in the top layer fluctuated around 10–12.5%, slightly above the estimated field capacity, with some peaks every other day due to watering events. Soil moisture in the middle layer was more stable than in the top layers and remained at a relatively constant value of approximately 12.5% for the entire growing seasons of 2012 and 2013. The first year, 2011, differed slightly from the other years (Figure S1). In this year, the middle soil layer dried out until July, probably due to transpiration from the high leaf biomass (as discussed below), but stabilized later at a value of approximately 10% after water supply was increased.

In contrast, notable wetting–drying cycles appeared in the extreme rainfall in both the top and middle soil layers in all years. Large water flushes replenished soil moisture in the top layer to values around 12.5–15% approximately every 4 weeks. The top soil dried out quickly and soil moisture declined steadily along the drought period to a minimum value of 2.5% for much of the season, well below the wilting point. The value of 2.5% is the minimum value that the soil moisture probes used were able to measure. Large wetting–drying cycles were also observed in the middle soil layer under extreme rainfall. The middle soil layer also dried out steadily during the drought period, reaching values below field capacity, but at lower pace than the top layer. Soil moisture measurements made in the top soil layer with a portable sensor confirmed the significant differences between rainfall treatments (Table S1, Figure S2). These differences were similar for all monocultures and the mixture (rainfall × plant community interaction *p* > 0.38 in all years).

Averaging over all five plant communities and the two rainfall treatments, a range of 14.3–21.5% of the water supplied in the growing season was lost in drainage (Table 1; Figure S3). Rainfall treatment had a significant effect on drainage in all years (*p* < 0.001; Table S2), with drainage being larger in regular than in extreme rainfall in the first year; and the reverse in subsequent years. Initially, drainage was independent of plant community. In 2013, a significant plant community × rainfall interaction was found (Table S2), as a result of larger drainage in extreme than in regular rainfall in the monocultures of the dicots *C. jacea* and *P. lanceolata* and in the mixture, but there were no differences between rainfall treatments in the monocultures of the two grasses (Figure S3).

### Responses of aboveground and belowground biomass to extreme rainfall treatment

**Fig. 2:**
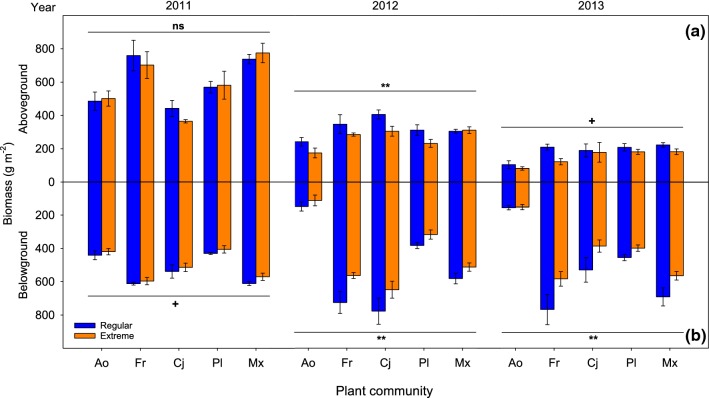
Aboveground (**a**) and belowground (**b**) biomass in the five communities (four monocultures and one mixture) under regular and extreme rainfall, at the end of each growing season in 2011, 2012, and 2013. Symbols indicate significant effect of rainfall within each year (see Table 2). Values are mean ± SE. ^ns^*p* > 0.1, ^+^*p* < 0.1, **p < 0.01. Ao, *Anthoxanthum odoratum*; Fr, *Festuca rubra*; Cj, *Centaurea jacea*; Pl, *Plantago lanceolata*; Mx, mixture

Aboveground biomass was much higher in the first year of the experiment than in later years. Also, effects of extreme rainfall on aboveground biomass varied over the years, with no effect in the first year (2011, *p* = 0.52) and the strongest effect in the second year (2012, *p* = 0.001) (Table 2a). Averaging across the five plant communities and the last 2 years of the experiment, aboveground biomass was 15.2% reduced in extreme rainfall compared to regular rainfall (Fig. 2). Importantly, rainfall effects were similar in all monocultures and the mixture (i.e., there was no significant plant community × rainfall interaction in any of the years).

**Table 2:** Results of analyses of variance (ANOVAs), split per year, for aboveground and belowground biomass with, (a) plant community (four monocultures and one mixture) and rainfall (regular versus extreme) as factors, and (b) diversity (observed mixtures versus expected mixtures) and rainfall as factors

Variable	Effect	2011			2012			2013		
		*df*	*F*	*p* value	*df*	*F*	*p* value	*df*	*F*	*p* value
(a) Aboveground biomass	Community (C)	4	17.86	< 0.001	4	7.44	< 0.001	4	5.89	0.001
	Rainfall (R)	1	0.42	0.522	1	12.15	0.001	1	3.64	0.064
	C × R	4	0.49	0.746	4	1.52	0.215	4	0.22	0.928
	Error	38			38			38		
Belowground biomass	Community (C)	4	30.57	< 0.001	4	71.034	< 0.001	4	88.35	< 0.001
	Rainfall (R)	1	3.12	0.085	1	11.00	0.002	1	10.94	0.002
	C × R	4	0.12	0.974	4	0.22	0.926	4	0.65	0.630
	Error	38			38			38		
(b) Aboveground biomass	Diversity (D)	1	16.01	< 0.001	1	1.16	0.294	1	8.10	0.010
	Rainfall (R)	1	0.01	0.918	1	3.59	0.073	1	4.64	0.044
	D × R	1	0.39	0.541	1	4.98	0.037	1	0.11	0.740
	Error	20			20			20		
Belowground biomass	Diversity (D)	1	23.10	< 0.001	1	8.33	0.009	1	30.96	< 0.001
	Rainfall (R)	1	2.43	0.134	1	7.55	0.12	1	9.32	0.006
	D × R	1	0.24	0.632	1	0.24	0.631	1	0.04	0.836
	Error	20			20			20		

Comparison of observed values versus expected values in mixtures is a common procedure to test overyielding

Extreme rainfall also affected root biomass with significant reductions in the second and third year of the experiment (Fig. 2; Table 2a). As with aboveground biomass, rainfall effects were similar for all four monocultures and the mixture, as revealed by the lack of significant plant community × rainfall interaction in all years (*p* > 0.6). Pooling over the four monocultures and the mixture and the last 2 years of the experiment, belowground biomass was reduced 17.4% in extreme rainfall compared to the regular water treatment.

### Diversity effects on aboveground and belowground biomass

**Fig. 3:**
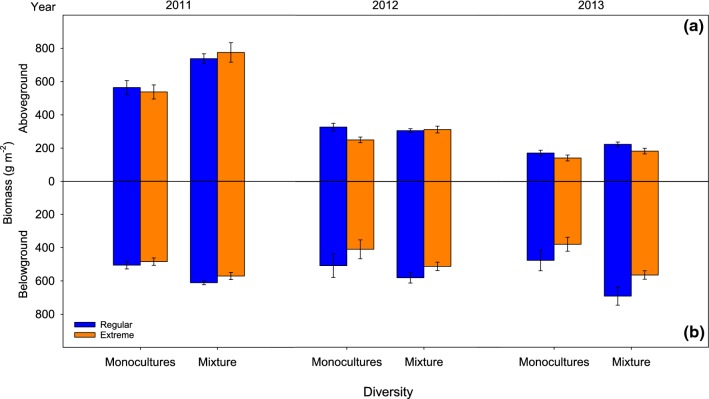
Aboveground (**a**) and belowground (**b**) biomass in the averaged four monocultures and the mixture, under regular and extreme rainfall, at the end of each growing season in 2011, 2012, and 2013. Values are mean ± SE

Regardless of the rainfall treatment, there was significantly higher shoot and root biomass in the mixture than expected from monocultures in the 3 years (i.e., aboveground and belowground overyielding) (Fig. 3). This was shown by the significant main effect of diversity and the lack of significance of the diversity × rainfall interaction when observed values in the mixture are compared with expected values from monocultures (Table 2b). An exception was 2012 (i.e., the second year), where this interaction was significant. In this year, shoot biomass of the mixture was not reduced by extreme rainfall, while monoculture biomass was reduced by 24%. Overall, the mixture produced 27.2% (2011), 19.9% (2012), and 23.0% (2013) more shoot biomass, and 16.2% (2011), 16.0% (2012), and 31.7% (2013) more root biomass, than expected from monocultures (Fig. 3).

There were shifts in species dominance, expressed as aboveground biomass, in the mixture aboveground and between years (Figure S4). Aboveground, the grass species *F. rubra* was overly dominant in the first year (2011), also dominant in the second year (2012), but became more equally abundant to both dicot species in the third year (2013). The grass *A. odoratum* gradually decreased in abundance in the second and third year of the experiment. These shifts in species dominance aboveground were similar for both rainfall treatments (species × rainfall interaction, *p* > 0.15 in all cases). Similar differences between species biomass and shifts over time without the effects of rainfall occurred belowground (root biomass determined sensu Mommer et al. (2008); results not shown).

### Responses of root distribution to extreme rainfall

**Fig. 4:**
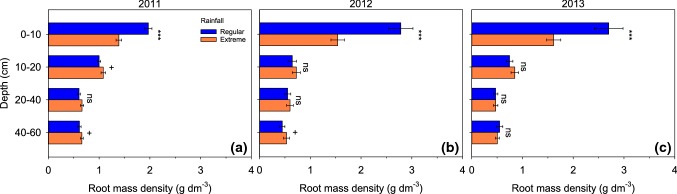
Root mass density per soil layer under regular and extreme rainfall, at the end of each growing season in 2011 (**a**), 2012 (**b**), and 2013 (**c**). The five plant communities (four monocultures and one mixture) were pooled because of the absence of significant effects of rainfall on plant community (ANOVA plant community × rainfall, *p* > 0.6, Table 3). Symbols indicate differences between rainfall treatments within each layer and year (see Table 4). Values are mean ± SE. ^ns^*p* > 0.1, ^+^*p* < 0.1, ***p < 0.001

**Fig. 5:**
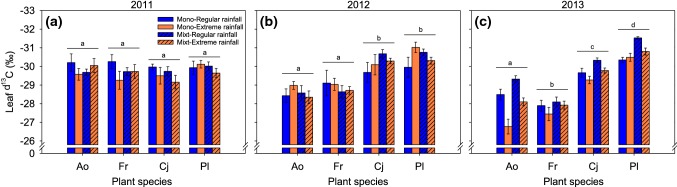
Leaf δ^13^C of each plant species (Ao, Fr, Cj, and Pl) at the end of each growing season in 2011 (**a**), 2012 (**b**), and 2013 (**c**), in monoculture (solid bars) and mixture (hatched bars) under regular (blue bars) and extreme (orange bars) rainfall. Note that taller bars (more negative δ^13^C) indicate lower water-use efficiencies. Different lower-case letters show significant differences between species within each year at *p* < 0.05. Values are mean ± SE. Ao, *Anthoxanthum odoratum*; Fr, *Festuca rubra*; Cj, *Centaurea jacea*; Pl, *Plantago lanceolata*. This figure is available in color in the online version of the journal (colour figure online)

The overall decrease in root biomass in all five plant communities was the result of a considerably reduced root mass density in the top soil under extreme rainfall, in all 3 years of the study (rainfall × depth interaction *p* < 0.001; Table 3a). On average, root mass in the top layer (0–10 cm depth) was reduced by 29.5% in 2011, 44.7% in 2012, and 40.2% in 2013, compared to regular rainfall (Fig. 4). Root mass density in deeper soil layers was similar in both rainfall treatments (Fig. 4), with the exception of a marginally significant higher root density in the bottom soil layer (40–60 cm depth) under extreme rainfall in 2011 (*p* = 0.087) and 2012 (*p* = 0.097). The vertical root distributions of all five plant communities (four monocultures and the mixture) responded similarly to the rainfall treatments (Fig. 5S), as indicated by the non-significant community × rainfall (*p* > 0.61) and the community × rainfall × depth interactions (*p* > 0.43) (Table 3a). Within the mixtures, the root distributions of the plant species responded to watering in a similar way as root distributions in monoculture (results not shown).

**Table 3:** Results of analyses of variance (ANOVAs), split per year, for root mass density with, (a) plant community (four monocultures and one mixture), rainfall (regular versus extreme), and soil depth (four soil layers) as factors, and (b) diversity (observed mixtures versus expected mixtures), rainfall (regular versus extreme), and soil depth (four soil layers) as factors

Effect	2011			2012			2013		
	*df*	*F*	*p*	*df*	*F*	*p*	*df*	*F*	*p*
(a)									
Community (C)	4	56.09	< 0.001	4	151.97	< 0.001	4	162.99	< 0.001
Rainfall (R)	1	7.84	0.006	1	15.82	< 0.001	1	6.13	0.014
Depth (D)	3	547.24	< 0.001	3	324.82	< 0.001	3	265.66	< 0.001
C × R	4	0.23	0.923	4	0.45	0.774	4	0.68	0.605
C × D	12	3.92	< 0.001	12	7.39	< 0.001	12	2.96	0.001
R × D	3	39.85	< 0.001	3	37.90	< 0.001	3	8.31	< 0.001
C × R × D	12	1.03	0.427	12	0.61	0.828	12	0.62	0.827
Error	152			152			152		
(b)									
Diversity (Dv)	1	44.49	< 0.001	1	10.48	0.002	1	57.70	< 0.001
Rainfall (R)	1	15.45	< 0.001	1	7.30	0.008	1	4.45	0.038
Depth (D)	3	341.55	< 0.001	3	220.23	< 0.001	3	207.15	< 0.001
Dv × R	1	0.90	0.347	1	0.50	0.484	1	0.57	0.451
Dv × D	3	3.87	0.012	3	8.362	< 0.001	3	0.81	0.49
R × D	3	37.88	< 0.001	3	25.14	< 0.001	3	9.36	< 0.001
Dv × R × D	3	1.84	0.146	3	0.12	0.949	3	0.37	0.772
Error	80			80			80		

Comparison of observed values versus expected values in mixtures is a common procedure to test diversity effects in mixtures

Root distribution responses to extreme rainfall were similar for the mixture and the monocultures, as the diversity × rainfall and diversity × rainfall × depth interactions were not significant in any of the years (Table 3b). The mixture had higher root mass density than expected from monocultures (highly significant diversity main effect; Table 3b), particularly so in the first three soil layers (until 40 cm depth).

### Leaf δ^13^C and water-use efficiency

Effects of rainfall on leaf *δ*^13^C built up over the 3 years of the experiment (Fig. 5; Table 4). Only in the last year, 2013, species had distinctively higher water-use efficiency (less negative leaf δ^13^C) in extreme rainfall than in regular rainfall, and lower WUE (more negative leaf δ^13^C) in the mixture than in monocultures. Large differences in leaf δ^13^C values were found between species, with shallower-rooted grasses (*A. odoratum* and *F. rubra*) having a higher water-use efficiency (significantly less negative leaf δ^13^C values), than the deeper-rooted dicots (*C. jacea* and *P. lanceolata*) (Table 4; Fig. 5). In the year 2013, the species x rainfall interaction was highly significant, particularly because *A. odoratum* increased WUE in extreme rainfall to a greater extent than the other species (Fig. 5).

**Table 4:** Results of analyses of variance (ANOVAs), split per year, for leaf δ^13^C, with plant species (four species), diversity (monoculture versus mixture), and rainfall (regular versus extreme) as factors

Effect	2011			2012			2013		
	*df*	*F*	*p*	*df*	*F*	*p*	*df*	*F*	*p*
Species (Sp)	3	0.78	0.507	3	30.06	< 0.001	3	142.96	< 0.001
Rainfall (R)	1	3.31	0.072	1	0.51	0.477	1	29.75	< 0.001
Diversity (Dv)	1	0.63	0.431	1	0.01	0.992	1	34.71	< 0.001
Sp × R	3	0.46	0.714	3	0.17	0.914	3	6.00	0.001
Sp × Dv	3	0.15	0.932	3	1.60	0.197	3	1.82	0.151
R × Dv	1	0.96	0.331	1	4.53	0.036	1	0.08	0.782
Sp × R × Dv	3	1.34	0.269	3	0.95	0.419	3	1.70	0.174
Error	80			80			78		

WUE responses to rainfall were independent of whether the plants were growing in monocultures or in the mixture in the years 2011 and 2013 (non-significant rainfall × diversity interaction; Table 4). This interaction was slightly significant in 2012, where species responded to extreme rainfall with slightly more negative leaf δ^13^C values in monocultures, but less negative values in mixtures.

## Discussion

Our experiment tested plant community responses to predicted climate change: the combination of extreme rainfall and drought. We varied the frequency of rainfall within the season, not the total amount of watering between the treatments. The extreme rainfall regime led to repeated periods of drought throughout the growing season, compared to the regular water regime. Intermittent heavy rainfall was not enough to fully buffer the negative effect of the drought, with an overall reduction in aboveground biomass of 15% over the last 2 years of the experiment compared to regular watering. This was accompanied by larger changes belowground with over 40% less root mass in the top soil layer where drying was most extreme. This effect was not different for monocultures compared to the mixture. Here, we discuss the possible mechanisms behind these findings and the consequences for the effects of biodiversity on mitigating climate change responses.

### How plant communities buffer within-season rainfall fluctuation

As typical for grasslands, a major part of the roots of all species was placed in the top soil layer (Ravenek et al. 2014; Schenk and Jackson 2002). Continuous soil water content measurements (Fig. 1) suggest that the water supplied was quickly taken up and transpired by the plant or evaporated from the soil, as shown in other experiments (Leimer et al. 2014; Milcu et al. 2016). Immediately in the first year of the experiment under extreme rainfall, on average 40% less roots were produced in the top soil layers, irrespective of plant species or diversity. Even the fine-rooted grasses with the highest capacity for water uptake from the top soil layers (Leimer et al. 2014) did not have the capacity to take up all the water supplied during the extreme rain events, given the water replenishment of the middle soil layer and increased leakage after the rainfall events (Fig. 1). Dense rooting in the top layer was constrained by the drought periods and the roots present might not have taken up much water during these periods (but see Prechsl et al. 2015).

We expected that under extreme watering, the plants would form more roots in middle and deeper soil layers to which some of the water would percolate. However, this did not occur. One reason may be that the water deficit may have been so intense during the summer that plants did not have enough resources to develop more roots. Another reason may be that the middle soil layers also moderately dried out during the drought periods (Figs. 1 and S1). Averaging over the growing season, soil moisture of the middle layer was 12.5% in the regular rainfall and 8.8% in the extreme rainfall in 2012, and of 13.0% and 9.4%, respectively, in 2013. This finding corroborates the conceptual framework established by Knapp et al. (2008) who modeled that larger and fewer rainfall events would reduce soil moisture in mesic ecosystems, thus leading to reduced plant productivity. Despite the replenishment of the middle and deeper layers by extreme rain events, plants may, thus, not have been able to develop high rooting densities in these middle and deep soil layers due an important cue for increased root growth and branching (i.e., higher soil moisture; de Kroon et al. 2009; Hutchings and de Kroon 1994; Wang et al. 2009) was lacking here.

How the water content of deeper soil layers develops under drought periods and rewetting depends on the exact rainfall regime, water holding capacity of the soil, and root profile. The presence or absence of deeper soil layers that are sufficiently moist may explain why, in some experiments, increased deeper rooting and water extraction did occur (Fort et al. 2017; Guderle et al. 2018; Hoekstra et al. 2015), but in others were not observed (Prechsl et al. 2015; this experiment). In all, with prolonged droughts and heavy rainfall events, plant communities may develop less biomass because deeper soil layers are only moderately wetted from which plants cannot sufficiently profit or leak to even deeper soil that is out of reach of plant roots.

These responses belowground were very similar for all species. Tall deep-rooting forbs have been shown to be more responsive in root architecture under water shortage (Guderle et al. 2018; Hoekstra et al. 2015; Leimer et al. 2014), but the two tall forb species in our experiment (*P. lanceolata* and *C. jacea*) did not respond any differently from the grasses. In an earlier pot experiment with eight species from temperate grasslands, we also found that the roots of all species responded in the same way to altered frequency of water supply, regardless of functional group (grasses versus dicots) (Padilla et al. 2013).

In addition to investing less in shallow roots, plants survived the drought and water loss under the extreme watering treatment by elevating water-use efficiencies. Illustrative is that, over time, all species tended to develop higher WUE under extreme watering (less negative leaf δ^13^C values; Fig. 5). Higher WUE reflects higher carbon production per unit of water loss due to reduced stomatal opening under water stress, which is typically a response to drought (Moreno-Gutiérrez et al. 2012; Padilla et al. 2013; Yin et al. 2005). Remarkably, it took until the last year before the effects of rainfall treatment on leaf δ^13^C values were significant, a delayed plastic response. The elevated WUE in the grasses also occurred in the last year. It is not exactly clear why this response took time to build up. Perhaps changes in leaf δ^13^C values were not only tied to water shortage but also to reduced growth and this may be why WUE responses were not seen in the first year. The first year was exceptional with high productivity due to the nutrient flush in freshly sieved soils, a common growth reaction in these experiments (Mommer et al. 2010). This high biomass production apparently also masked an aboveground biomass response to the water treatments in the first year, although belowground the root placement responded immediately.

In conclusion, our four species responded very similarly to the extreme rainfall treatment that we imposed, despite that grassland species typically can have quite different traits by which they vary in their responses to drought (Elst et al. 2017; Guderle et al. 2018), water uptake from different soil layers (Fort et al. 2017; Hoekstra et al. 2015), and affecting soil structure and water distribution (Fischer et al. 2015, 2019). One reason may be that these four species were simply not different enough in this respect, despite the fact that they belonged to two very different functional groups. On the other hand, the similarity in response is consistent with recent studies showing that complementarity in water use between species and functional groups is limited (Bachmann et al. 2015; Jesch et al. 2018). Species may be less able to differentiate in the use of water resources in space and time, than in the use of other resources.

### No biodiversity effect in response to within-season rainfall fluctuation

In line with the similar responses of each of the species, the four-species mixture did not buffer the extreme rainfall treatment in another way than the monocultures. This is a surprising result, in light of the widely observed positive effects of diversity in response to climatic extremes (Isbell et al. 2015; Tilman and Downing 1994; Wagg et al. 2017; Wright et al. 2015), and assuming that the results of our four-species mixtures versus monocultures are representative for biodiversity effects in general. As in an earlier experiment with a similar four-species mixture (Mommer et al. 2010), the mixture produced more aboveground and belowground biomass than was expected from the respective monocultures (overyielding). This biodiversity effect appeared similar under both rainfall regimes in our experiment. Hofer et al. (2016) also demonstrated overyielding independent of rainfall. There is increasing evidence that the release of species-specific pathogenic soil biota in species-rich communities (de Kroon et al. 2012; Hendriks et al. 2013; Mommer et al. 2018) plays an important role in overyielding, although other factors may play a role as well (Barry et al. 2019). These factors seem unaffected by rainfall.

Wagg et al. (2017) showed that more diverse plant communities were better able to buffer summer droughts by their enhanced productivity in the spring, when water was not yet limiting. This led to enhanced variation within the season and long-term stability. The water regime that we imposed with our extreme rainfall treatment would not allow for such early-season overyielding preceding the responses to rainfall frequency, as we altered rainfall frequency already from April onwards. What other factors can explain the overall lack of diversity on the response to rainfall regime? On one hand, the mixture seems to have experienced less water stress, evidenced by the lower water-use efficiencies of the plants in mixture compared to those in monoculture, despite the drought periods in the extreme rainfall treatment. These results are consistent with those of Van Peer et al. (2004) and Guderle et al. (2018) demonstrating higher stomatal conductance and, thus, lower water-use efficiency, in more diverse plant communities, at least for some functional groups. On the other hand, the higher aboveground biomass of more species-rich communities also results in higher total leaf transpiration (Guderle et al. 2018; Leimer et al. 2014; Milcu et al. 2016) and, thus, faster depletion of soil water. In addition, since species-rich vegetation leads to a densely rooted top soil layer (Mommer et al. 2010; Ravenek et al. 2014) and a lower soil water retention capacity (Fischer et al. 2015, 2019), these communities may be less resistant with stronger biomass reduction to rainfall fluctuations, particularly if the deeper rooting species still cannot access soil water and buffer those effects.

As we only changed the frequency of watering and not the total amount, within each separate year, our results raise the question whether buffering of extremes by more diverse communities due to the insurance effect only takes place in situations of extended resource shortage. Biodiversity enhances ecosystem functionality particularly in environments of major resource shortage such as global drylands (Maestre et al. 2012). Grossiord et al. (2014), reconstructing responses to drought in European forest stands, concluded that higher tree diversity enhances resistance to drought events only in drought-prone environments. When extreme situations occur but overall resources stay the same, or are enhanced as after flooding (Wright et al. 2015), the diversity-stability hypothesis may not be as straightforward as originally anticipated. More work is needed on how stress responses of species interact with their resource utilization (Pfisterer and Schmid 2002; Van Peer et al. 2004; Wright et al. 2015, 2017), and how these combined responses determine the biodiversity effects on ecosystem functioning to environmental extremes.

## Electronic supplementary material

Below is the link to the electronic supplementary material.

Supplementary material 1 (DOCX 1304 kb)
