# Supplementary material for: Effects of extreme rainfall events are independent of plant species richness in an experimental grassland community
Source: Oecologia. 2019 Aug 10;191(1):177–90. doi: 10.1007/s00442-019-04476-z (PMC6732129; doi:10.1007/s00442-019-04476-z)
Supplement: Supplementary file 1 — Supplementary material 1 (DOCX 1304 kb) [file 442_2019_4476_MOESM1_ESM.docx]

**Electronic Supplementary Material**

**Resistance to extreme rainfall fluctuation is independent of plant species richness in an experimental grassland community**

Francisco M. Padilla, Liesje Mommer, Hannie de Caluwe, Annemiek E. Smit-Tiekstra, Eric J.W. Visser, Hans de Kroon

*Determination of rainfall treatments from analysis of precipitation patterns*

The amount and frequency of rainfall was based on the precipitation record over 1975-2009 of the nearest meteorological station (Volkel, 51° 39' N, 05° 42' W, 20 metres above sae level, KNMI; 23 km from the Nijmegen Phytotron). Analysis of this record revealed that rainfall (716±20 mm year^-1^) was not concentrated in a few rain events, months or seasons, but it fell in a rather frequent pattern, with little differences between months and seasons. On a yearly basis, rain spells (> 1mm) of one-day duration were the most frequent rain events (50% of the events). As for the duration of dry periods interspersed between two rain events, drought spells of one-day duration were the most frequent (34% of the droughts). Based on this account, the regular rainfall treatment consisted of one watering event every other day; 15 watering events were applied on a monthly basis. The averaged monthly rainfall in Volkel (60±20 mm) served as a reference for the amount of water supplied. However, rainfall was increased nearly 1.5 times in the first year because soils dried out quickly to wilting point values in the first growing season due to high water uptake by the high aboveground biomass. Average watering was 93.4 mm month^-1^ (6.2 mm each watering) in the growing season of 2011 and was reduced to 66.3 and 57.9 mm month^-1^ in the growing seasons of 2012 and 2013, respectively, giving an average watering amount per event of 4.8 and 4.7 mm, respectively (Table 1).

The extreme rainfall treatment aimed at resembling a climate change scenario for mid-western Europe of increased extreme rain events in the growing season, with decreasing the number of rain events and maintaining the mean amount of rainfall in summer ([Easterling *et al.*, 2000](#_ENREF_1), [IPCC, 2014](#_ENREF_2), [Kovats *et al.*, 2014](#_ENREF_3), [van der Hurk *et al.*, 2006](#_ENREF_4)). This treatment concentrated the same amount of watering supplied in the regular treatment, on a monthly basis, but in a few large watering events, thus imposing increased intensity of rainfalls and drought spells between rain events. The adopted extreme rainfall was obtained from the rainfall analysis of two extreme years in the Netherlands, characterized by long drought spells between heavy rains in spring and summer, assuming that these two years are likely to resemble the forecasted future rainfall scenarios for mid-western Europe. The precipitation record in Volkel showed very unusual drought spells of 20 up to 38 days in the growing seasons of years 2003 and 2007. Thus the extreme rainfall treatment consisted of one watering event every 31 days, on average, totalling the amount of watering provided in the regular rainfall treatment during that dry spell (Table 1). However, for practical reasons, at the end of each dry spell, watering was split and supplied in three consecutive days to prevent water spilling. On average, each of the three watering events for each dry spell was of 25.9 mm in 2011, 19.5 mm in 2012 and 21.4 mm in 2013. Rainfall treatments were applied only in the growing season (Mar-Apr to Sep), consistent with forecasted rainfall scenarios, with a total of six dry spells per growing season in 2011 and 2012, and five dry spells in 2013. In autumn and winter, the same regular watering was applied in both treatments. During the growing season, drainage from each unit was collected in separate bottles and measured every two days.

Table S1. Results of repeated-measures analysis of variance (RM-ANOVA), split per year, for soil moisture measurements made with a portable sensor (ThetaProbe ML2x, Delta-T Devices Ltd., Cambridge, UK) with plant community (four monocultures and one mixture) and rainfall (regular versus extreme) as between-subjects factors, and time (i.e., date of soil moisture measurement) as within-subjects factor. Depending on the year, there were between 17 and 23 soil moisture measurements (time points).

|  | 2011 |  |  |  | 2012 |  |  |  | 2013 |  |  |
| --- | --- | --- | --- | --- | --- | --- | --- | --- | --- | --- | --- |
| Effect | d.f. | *F* | *p-value* |  | d.f. | *F* | *p-value* |  | d.f. | *F* | *p-value* |
| Between-subjects |  |  |  |  |  |  |  |  |  |  |  |
| Community (C) | 4 | 1.43 | 0.244 |  | 4 | 2.46 | 0.062 |  | 4 | 7.11 | <0.001 |
| Rainfall (R) | 1 | 1792.89 | <0.001 |  | 1 | 1949.78 | <0.001 |  | 1 | 1349.56 | <0.001 |
| C x R | 4 | 0.17 | 0.955 |  | 4 | 1.064 | 0.388 |  | 4 | 0.63 | 0.641 |
| Error | 38 |  |  |  | 38 |  |  |  | 38 |  |  |
| Within-subjects |  |  |  |  |  |  |  |  |  |  |  |
| Time (T) | 3.14 | 297.36 | <0.001 |  | 8.23 | 469.46 | <0.001 |  | 7.54 | 111.43 | <0.001 |
| T x C | 12.57 | 1.19 | 0.297 |  | 32.9 | 2.84 | <0.001 |  | 30.18 | 1.19 | 0.053 |
| T x R | 3.14 | 91.78 | <0.001 |  | 8.23 | 304.49 | <0.001 |  | 7.54 | 185.14 | <0.001 |
| T x C x R | 12.57 | 0.58 | 0.860 |  | 52.77 | 1.39 | 0.083 |  | 30.18 | 1.47 | 0.060 |
| Error | 119.45 |  |  |  | 312.55 |  |  |  | 286.68 |  |  |

Table S2. Results of analysis of variance (ANOVA), split per year, for total percentage of rainfall lost through drainage per season, with plant community (four monocultures and one mixture) and rainfall (regular versus extreme) as factors.

|  | 2011 | | |  | 2012 | | | |  | | 2013 | | | |
| --- | --- | --- | --- | --- | --- | --- | --- | --- | --- | --- | --- | --- | --- | --- |
| Effect | d.f. | *F* | *p-value* |  | d.f. | *F* | *p-value* |  | | d.f. | | *F* | *p-value* |  |
| Community (C) | 4 | 3.22 | 0.023 |  | 4 | 17.04 | <0.001 |  | | 4 | | 10.50 | <0.001 |  |
| Rainfall (R) | 1 | 24.73 | <0.001 |  | 1 | 40.72 | <0.001 |  | | 1 | | 19.88 | <0.001 |  |
| C x R | 4 | 0.80 | 0.531 |  | 4 | 2.13 | 0.096 |  | | 4 | | 3.28 | 0.021 |  |
| Error | 37 |  |  |  | 38 |  |  |  | | 38 | |  |  |  |


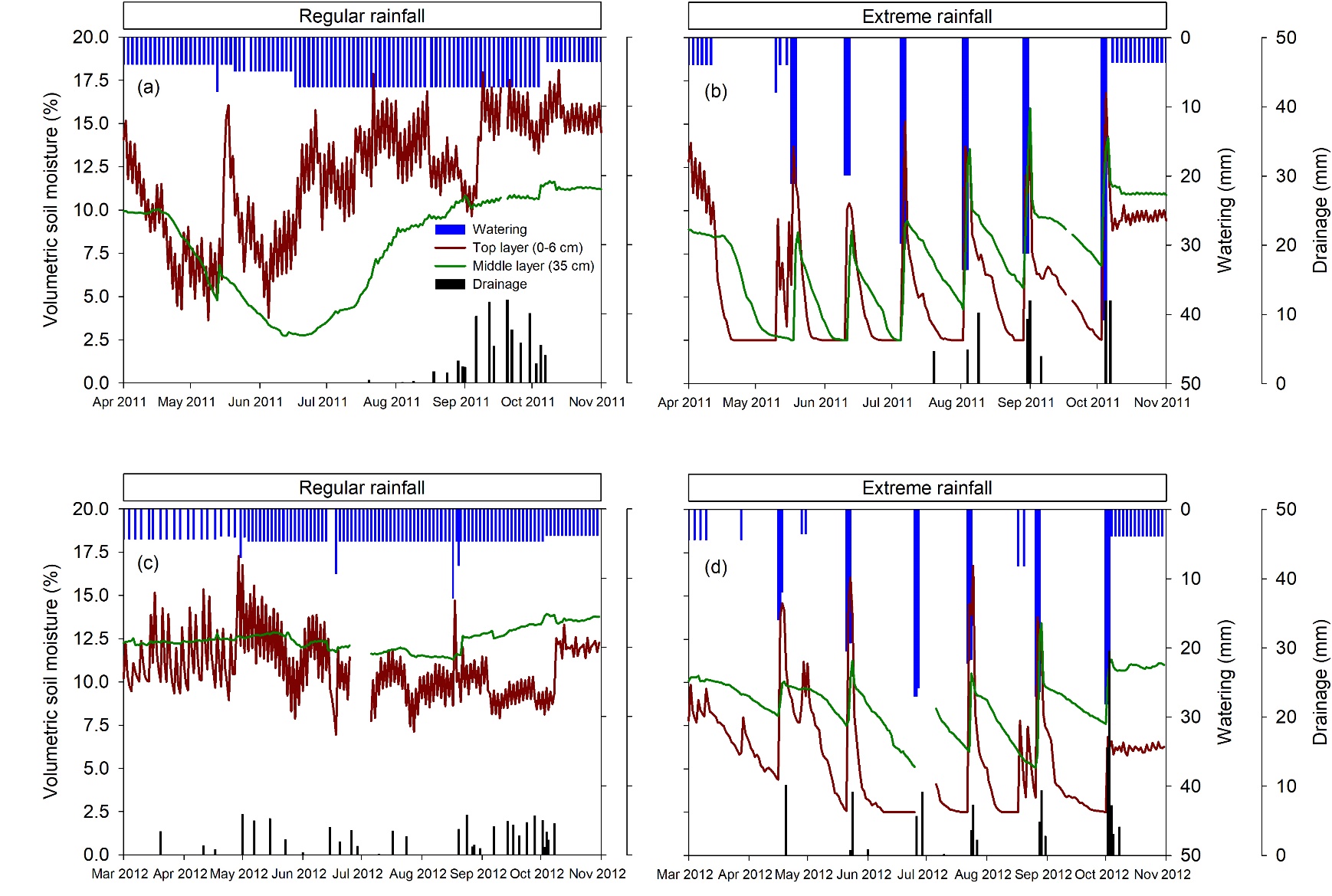
Figure S1. Soil moisture dynamics in the growing seasons of 2011, (a) and (b), and 2012, (c) and (d), in the top (0-6 cm depth) and middle (35 cm depth) soil layers, and watering and drainage, in the regular and extreme rainfall treatments. Soil moisture values are daily averages of one replicate of each of the four monocultures and two replicates for the mixture.

Figure S2. Soil moisture measurements, in the top soil layer (0-6 cm depth), in the growing seasons of 2011, 2012 and 2013, of the five plant communities (four monocultures and one mixture), in the regular and extreme rainfall treatments. Values are mean ± SE (*n*=4 in monocultures, 8 in mixtures).


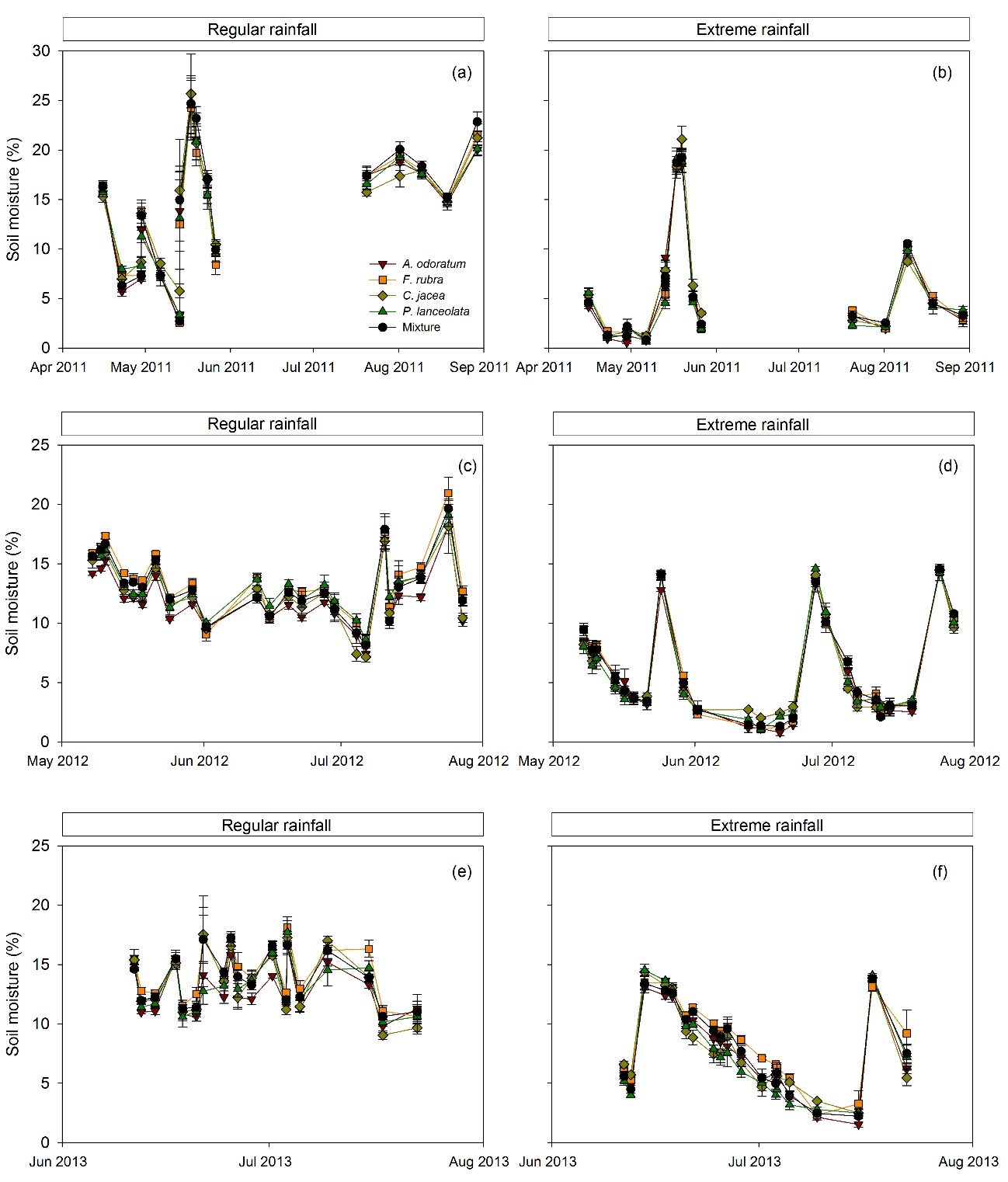


Figure S3. Percentage of rainfall lost through drainage in the five plant communities (four monocultures and one mixture) under regular (blue bars) and extreme (orange bars) rainfall, at the end of each growing season in 2011, 2012 and 2013. In 2011 and 2012, different letters in legends indicate significant effect of rainfall regardless of plant community because the community x rainfall interaction was not significant; in 2013, symbols indicate significant effect of rainfall for each community because of the significance of the community x rainfall interaction (see Table S2). Values are mean ± SE. ^ns^ p>0.1, ** p<0.01, *** p<0.001. Ao, *Anthoxanthum odoratum*; Fr, *Festuca rubra*; Cj, *Centaurea jacea*; Pl, *Plantago lanceolata*.


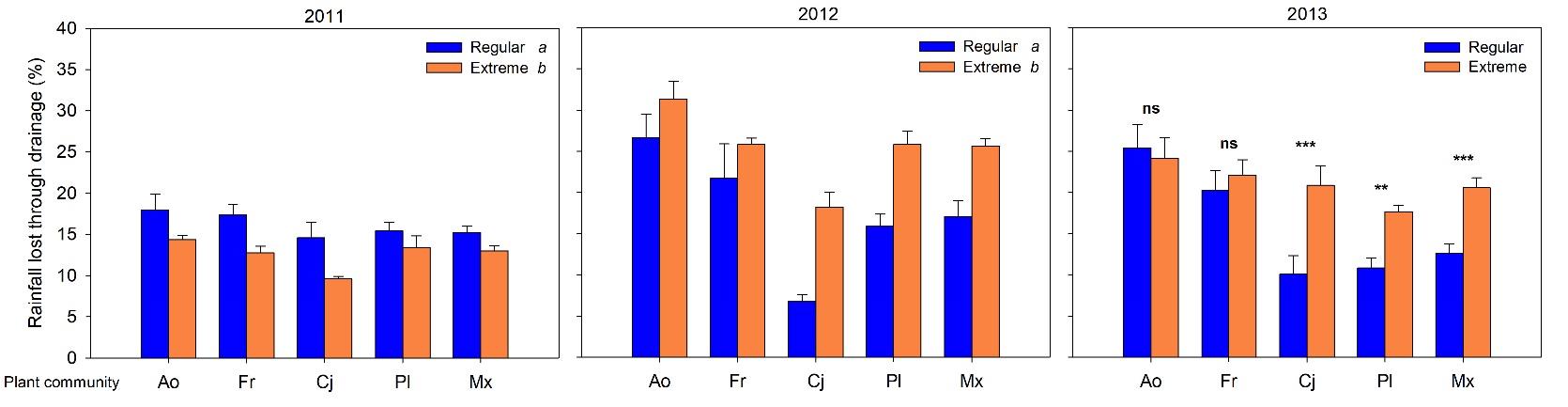


Figure S4. Aboveground biomass of each plant species (Ao, Fr, Cj and Pl) in the mixture community under regular (blue bars) and extreme (orange bars) rainfall, at the end of each growing season in 2011, 2012 and 2013. In each of the three years, there were very significant differences between species (ANOVA, p<0.001), regardless of the rainfall treatment (ANOVA, species x rainfall interaction p>0.15). ns shows no significant effect (p>0.23) of rainfall in each of the three years. Values are mean ± SE. Ao, *Anthoxanthum odoratum*; Fr, *Festuca rubra*; Cj, *Centaurea jacea*; Pl, *Plantago lanceolata*.


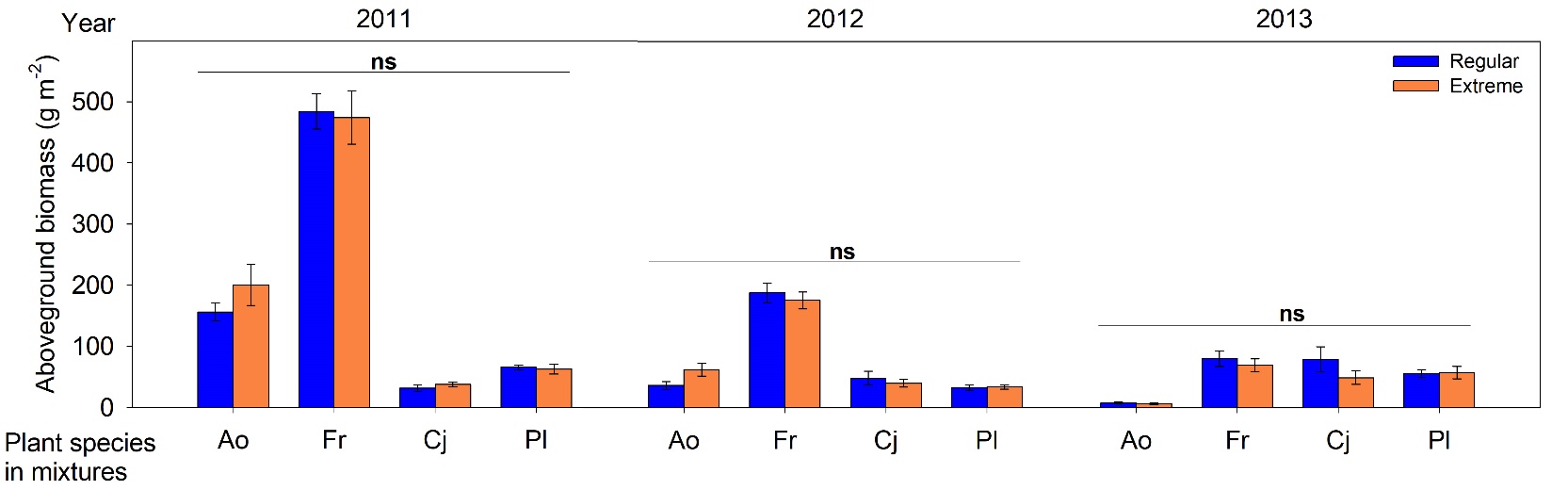


Figure S5. Root mass density per soil layer of each of the five plant communities under regular and extreme rainfall, at the end of each growing season in 2011, 2012 and 2013. Values are mean ± SE.


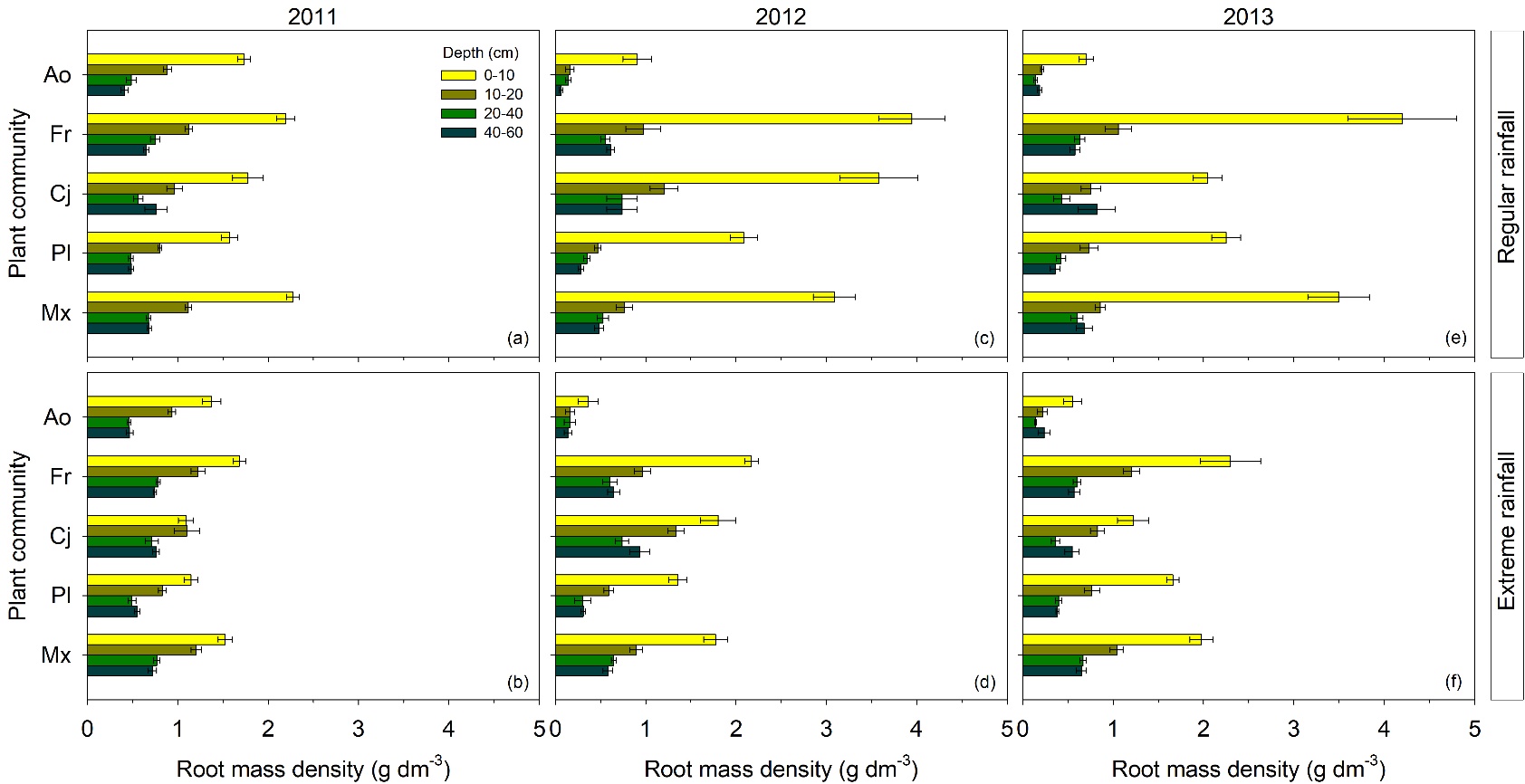


**References**

Easterling DR, Meehl GA, Parmesan C, Changnon SA, Karl TR, Mearns LO (2000) Climate extremes: Observations, modeling, and impacts. Science*,* **289**, 2068-2074.

IPCC (2014) *Climate Change 2014: Synthesis Report. Contribution of Working Groups I, II and III to the Fifth Assessment Report of the Intergovernmental Panel on Climate Change,* Geneve, Switzerland, IPCC.

Kovats RS, Valentini R, Bouwer LM *et al.* (2014) Europe. In: *Climate Change 2014: Impacts, Adaptation, and Vulnerability. Part B: Regional Aspects. Contribution of Working Group II to the Fifth Assessment Report of the Intergovernmental Panel on Climate Change.* (eds Barros VR, Field CB, Dokken DJ, Mastrandrea MD, Mach KJ, Bilir TE, Chatterjee M, Ebi KL, Estrada YO, Genova RC, Girma B, Kissel ES, Levy AN, Maccracken S, Mastrandrea PR, White LL) pp Page. Cambridge, United Kingdom and New York, NY, USA, Cambridge University Press.

Van Der Hurk B, Tank AK, Lenderink G *et al.* (2006) *KNMI Climate Change Scenarios 2006 for the Netherlands,* De Bilt, the Netherlands, KNMI.
